# Spectral Efficiency Improvement of 5G Massive MIMO Systems for High-Altitude Platform Stations by Using Triangular Lattice Arrays

**DOI:** 10.3390/s21093202

**Published:** 2021-05-05

**Authors:** Francesco Alessio Dicandia, Simone Genovesi

**Affiliations:** Dipartimento di Ingegneria dell’Informazione, University of Pisa, 56122 Pisa, Italy; simone.genovesi@unipi.it

**Keywords:** phased array, massive MIMO, 5G, wideband array, triangular grid

## Abstract

The beneficial effects of adopting a triangular lattice on phased arrays with regular and periodic grids for high-altitude platform station (HAPS) systems are presented in the scenario of massive MIMO communications operating within the 5G NR n257 and n258 frequency bands. Assessment of a planar array with 64 elements (8 × 8) is provided for both a triangular lattice and a square one in terms of array gain, average sidelobe level (ASLL), and mutual coupling. Particular attention is devoted to illustrating the impact of the antenna array lattice at the system level by evaluating its significant merits, such as its spectral efficiency (SE) and signal-to-interference ratio (SIR). The better performance exhibited by the triangular lattice array in comparison to the square one makes it appealing for the 5G massive MIMO paradigm.

## 1. Introduction

The upcoming next generation (5G) of wireless communication networks is expected to drastically improve overall system performance, such as data throughput and energy efficiency [1,2]. Currently, in most wireless communication systems, the users inside a sector cell are served through a base station, whose radiation pattern consists of a fixed broad main beam. In this scenario, the wireless communications system turns out to be inefficient from the energy point of view, since most of the base station (BS)’s radiated signal propagates towards directions in which there are no users [3]. The exploitation of larger frequency bandwidths and the deployment of more BSs to reduce the cell area are adopted in order to tackle the ever-increasing data throughput. On the other hand, in upcoming 5G wireless technology, improvement of the data throughput is guaranteed mainly by massive multiple-input and multiple-output (MIMO) technology [4,5,6], which is capable of serving multiple users simultaneously within the same time–frequency resource, through a multibeam radiation pattern with a consequent increase in the spectral efficiency (SE) of the system [2]. The coverage of users inside a sector cell is achieved through the deployment of phased arrays with a massive number of antennas. These arrays are able to provide advanced beamforming and beam tracking to generate multiple concurrent beams that send the different streams of data allocated on the same time–frequency resource to separate users [7]. In addition to radiation pattern optimization, a large-frequency spectrum is pivotal for supporting communication links with increased data transmission rates between the base station and the users. For this reason, the limited available spectrum below 6 GHz no longer satisfies the system’s needs; consequently, the millimeter-wave (mm-wave) band has recently drawn great attention for the next 5G wireless communications systems [8,9,10,11]. 

The design of massive MIMO phased array antennas represents one of the most challenging aspects to tackle in order to guarantee reliable performance. In fact, the array performance in terms of gain, beam steering, peak sidelobe level (PSLL), antenna mutual coupling, and inter-element distance directly affects the quality of the overall wireless communication. Some examples of antenna arrays for mm-wave communication are reported in Reference [12]. In Reference [13], a 28 GHz phased array composed of 64 elements (8 × 8) is described. Massive MIMO systems with 64 elements operating around 28 GHz and 40 GHz were designed with fully digital beamforming in References [14,15]. Dual-polarized phased array transceivers able to provide two concurrent independent beams, and hence double the channel capacity, have also been proposed [16,17].

**Figure 1:**
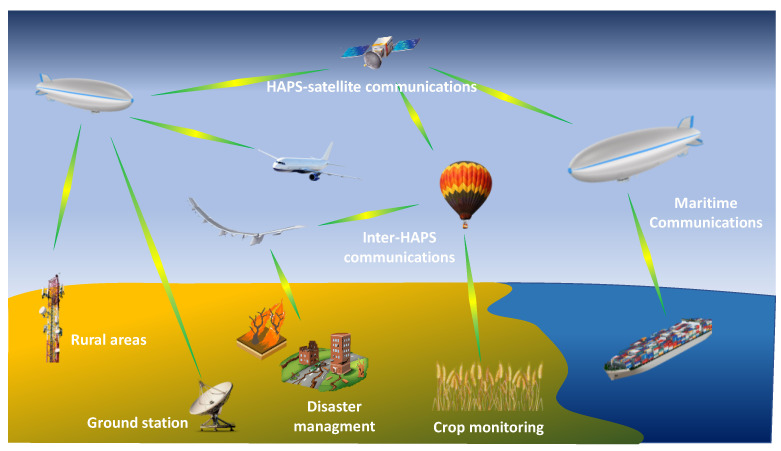
Examples of several scenarios where HAPSs can be exploited.

However, some obstacles prevent the achievement of both seamless and ubiquitous wireless connectivity if only the terrestrial infrastructure is considered. In fact, terrestrial ground stations cannot be deployed in off-grid or inaccessible areas, such as rural zones, oceans, deserts, and generally harsh and remote environments. To this end, aerial wireless communication based on the employment of high-altitude platform stations (HAPSs) will play a paramount role in providing everywhere with access to the global network [18,19,20]. A HAPS consists of an unmanned aerial vehicle (UAV)—such as a gas-filled balloon, airship, or aircraft—operating in the stratosphere at an altitude of around 20 km [21] (Figure 1). HAPSs can be deployed in wireless communication networks with different topologies, within which they act mainly as aerial relays or aerial base stations to help improve the wireless communication [19]. In the former case, a HAPS collaborates with a ground BS by offering an alternative reliable link between it and a ground user by forwarding the data in case of a blockage between them. In the latter case, the HAPS acts as an aerial base station by offering wide wireless connectivity between ground users and the core network in the event of an inadequate terrestrial network or temporary ground station malfunction or maintenance. Moreover, thanks to their rapid deployment, HAPSs can assist in readily deploying communication networks after catastrophic events, such as earthquakes [22,23]. Furthermore, HAPSs can act as reliable relays between terrestrial users and CubeSats [24,25]—low Earth orbit (LEO) satellites—in order to form an airborne communication network (ACN) [26,27]. 

The antenna system certainly represents one of the most important factors for HAPSs where reliable performance is concerned [28]. In Reference [29] a multibeam lens antenna for a HAPS operating at L/S band is presented. A Ka-band phased array composed of 256 open-ended substrate-integrated square waveguides and a 4-channel beamformer circuit produced by Anokiwave was described in Reference [30].

In addition to the above-mentioned critical phased array aspects, another meaningful parameter that has to be accurately investigated for large phased arrays at mm-wave is the thermal aspect [31]. From the electromagnetic perspective, the most straightforward way of improving the cooling performance to dissipate heat is to increase the distance between the array elements. However, increasing the minimum distance between the single radiators too much can degrade the PSLL, with the appearance of grating lobes inside the visible region. This is detrimental to the signal-to-interference ratio (SIR) in massive MIMO systems with multiuser communication within the same time–frequency resource. Recently, a solution based on aperiodic antenna element arrangement capable of reducing the PSLL inside a defined sector cell for massive MIMO systems has been proposed in Reference [32]. However, the array aperiodicity considerably increases the design complexity of the feeding network, as well as complicating the array calibration.

Most of the designed phased arrays with uniform lattices rely on square or rectangular element grids, although a triangular lattice allows for an increase in the minimum antenna distance, avoiding the onset of grating lobes [33,34]. Few examples of triangular lattice 5G phased arrays are reported in the literature [35,36]. However, a comprehensive analysis addressing the overall performance of massive MIMO systems, including SE and SIR, has not been yet presented, although some recent studies have proven the advantages of triangular lattice arrays over square and rectangular ones for 5G massive MIMO system ground stations [37].

The previous mm-wave 5G phased antenna arrays were designed to operate in one specific band, even if there are different frequency bands allocated to 5G mm-wave worldwide. For this reason, it is advantageous to design phased arrays that can operate over a wide band in order to allow for multistandard operation or exploit interband carrier aggregation (CA) to enhance spectral efficiency [38].

In this paper, the beneficial effects of adopting a triangular lattice rather than a square one are described and tested on a phased array for HAPS communications within both the 5G New Radio (NR) n258 (24.25–27.5 GHz) and NR n257 (26.5–29.5 GHz) bands. A thorough analysis of the overall system performance—including array gain, average sidelobe level (ASLL), signal-to-interference ratio (SIR), and spectral efficiency (SSE)—is carried out in order to demonstrate the effects of the employed array lattice. Furthermore, the comparison to a planar array of 8×8 elements in terms of array gain, the minimum distance between elements, and ASLL is presented in Section 2. Section 3 is devoted to highlighting the superior robustness of triangular lattices for the antenna elements’ impedance variation during beam steering inside the sector when compared to square lattices, via full-wave electromagnetic simulations. This represents a step forward with respect to Reference [37], since it better quantifies the effects of mutual coupling and matching efficiency (*η_Γ_*). Massive MIMO metrics, such as SE and SIR, are evaluated in Section 4, whilst conclusions are summarized in Section 5.

## 2. Triangular vs. Square Lattice Planar Arrays

The radiation pattern (RP) generated by a planar array with *N*×*M* elements in the event of uniform amplitude excitation, and when neglecting the mutual coupling, is equal to [39]:

(1) $$\begin{array}{l} \mathrm{RP}\left(\theta ,\phi \right)=\sum_{n=0}^{N-1}\sum_{m=0}^{M-1}e^{j\varphi_{nm}}E_{nm}\left(\theta ,\phi \right)\;e^{j\beta F}\\ F=x_{nm}\sin\left(\theta \right)\cos(\phi )+y_{nm}\sin(\theta )\sin(\phi ) \end{array}$$

where *β* *=* 2π*/λ*_0_ is the phase constant; *E_nm_*(*θ*,*ϕ*) represents the elements’ radiation pattern; and *φ_nm_* represents the phase associated with the (*n,m*)-*th* element necessary to steer the main beam toward the desired direction (*u_0_*, *v_0_*), which depends on the element’s position (*x_nm_*, *y_nm_*) within the employed lattice. The relation between the phase *φ_nm_* and the element’s position (*x_nm_*, *y_nm_*) is given by the following equation:

(2) $$\begin{array}{l} \varphi_{nm}=\beta \left(x_{nm}u_{0}+y_{nm}v_{0}\right)\\ u_{0}=\sin(\theta_{0})\cos(\phi_{0})\;\\ v_{0}=\sin(\theta_{0})\sin(\phi_{0}) \end{array}$$

The minimum distance between elements (*d*_0_) is a fundamental parameter for array radiation performance. Furthermore, *d*_0_ should be selected in order to avoid the appearance of grating lobes within the visible region during the beam steering inside the angular sector selected to serve the users. In fact, high lateral lobes do not only provide a considerable reduction in the maximum array gain, but also produce interference for all other users served within the same time–frequency resource. In general, the maximum value of the minimum distance between the antenna elements depends on the maximum steering angle necessary to cover the area of interest.

**Figure 2:**
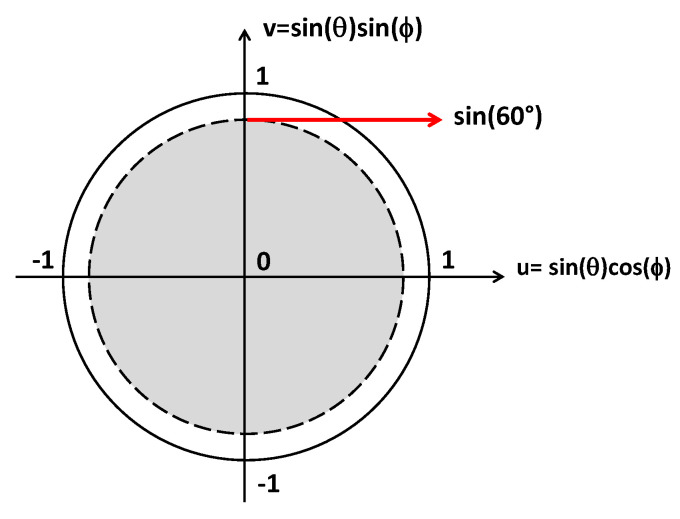
Circular sector cell in the *u–v* plane within which the HAPS antenna array has to steer the main beam to serve the 5G users.

The HAPS scenario differs from that of a 5G ground BS—whose coverage spans 30° in elevation and 120° in azimuth [37,40]—since a circular scan area turns out to be more appropriate [18]. Therefore, it is assumed to cover an angular sector with a maximum steering angle of 60° off broadside (–60 ≤ *θ*_0_ ≤ 60°) for all *ϕ* angles (0 ≤ *ϕ*_0_ ≤ 180°), as shown in Figure 2 in the *u–v* plane.

As stated before, most of the designed phased arrays with uniform lattices rely upon a square arrangement of the antenna elements. In this case, the condition on *d*_0_ for avoiding the appearance of grating lobes inside the visible region is given by:

(3) $$d_{0}\leq \frac{\lambda_{HF}}{R+\sin\left(\theta_{\max}\right)}$$

where λ_HF_ represents the wavelength evaluated at the highest frequency (i.e., 29.5 GHz); *θ*_max_ is the maximum antenna array steering angle, which is 60° for the considered circular angular sector shown in Figure 2; and *R* is a real number that represents the distance in the *u–v* plane between the closest grating lobes and the center of the visible region (*u* = 0, *v* = 0). Therefore, in order to guarantee the absence of undesirable high lobes inside the visible region, *R* has to be set to greater than 1.

**Figure 3:**
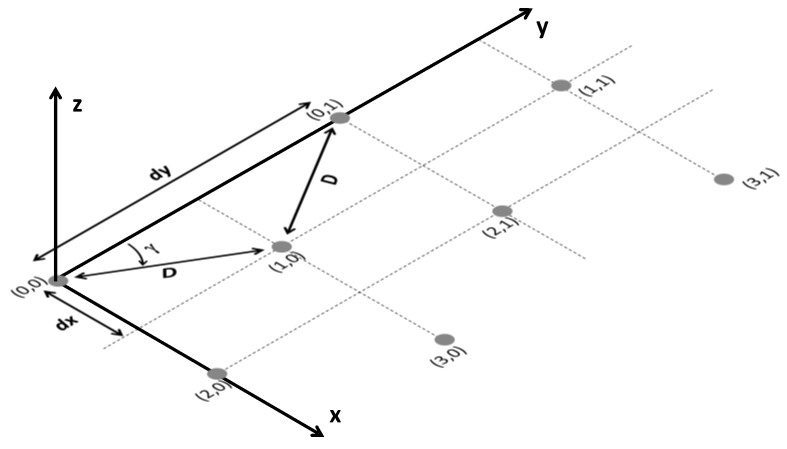
Planar antenna array with a triangular lattice.

In the case of an antenna array with uniform spacing but with a triangular grid, the spacing *d_x_* and *d_y_*—namely, the height and the base of the triangle (Figure 3)—are related to the *γ* angle by the following equations:

(4) $$\begin{array}{l} d_{x}=D\sin\left(\gamma \right)\\ d_{y}=2D\cos\left(\gamma \right) \end{array}$$

where *D* is the distance between the (*n,m*)-*th* element and the (*n*+1*,m*)-*th*, as depicted in Figure 3.

**Figure 4:**
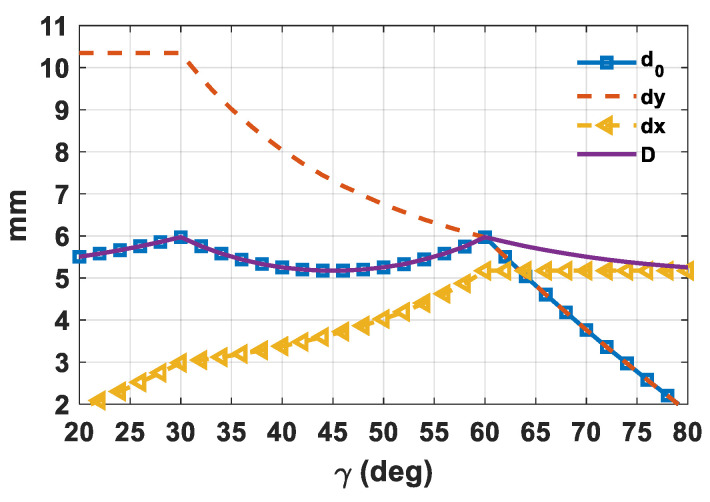
Elements’ spacing (*d_x_*, *d_y_*), the antenna elements’ distance (*D*), and its minimum (*d*_0_) as a function of the γ value for a triangular lattice array in the event of *R* = 1.1.

Differently from a square lattice, there are different choices of grid spacing (*d_x_*, *d_y_*) in an array with a triangular grid, which are able to guarantee the absence of grating lobes. In this case the grating lobes are avoided if the following equations are satisfied:

(5) $$\begin{array}{l} {\left(\frac{\lambda_{HF}}{2d_{x}}-u_{0}^{*}\right)}^{2}+{\left(\frac{\lambda_{HF}}{d_{y}}-v_{0}^{*}\right)}^{2}\geq R^{2}\\ \frac{2\lambda_{HF}}{d_{y}}-\sin\left(\theta_{\max}\right)\geq R\\ \frac{\lambda_{HF}}{d_{x}}-\sin\left(\theta_{\max}\right)\geq R\\ u_{0}^{*}=\sin\left(\theta_{\max}\right)\cos\left(\gamma \right)\\ v_{0}^{*}=\sin\left(\theta_{\max}\right)\sin\left(\gamma \right) \end{array}$$

where u_0_^*^ and v_0_^*^ represent the *u–v* plane coordinates where the grating lobes can appear. It is worthwhile to note that the previous grating lobes absence equations are only valid for equilateral or isosceles triangular lattices. Scalene lattices have not been considered since they provide a lower value for *d*_0_. Equation (5) was used to understand the effect of *γ* on the element spacing in a circular scan sector. The elements’ spacing (*d_x_*, *d_y_*), along with the minimum distance between them, were evaluated for *γ* within the interval [20°,80°] in the event of *R* = 1.1. The plot summarizing the results is shown in Figure 4.

**Figure 5:**
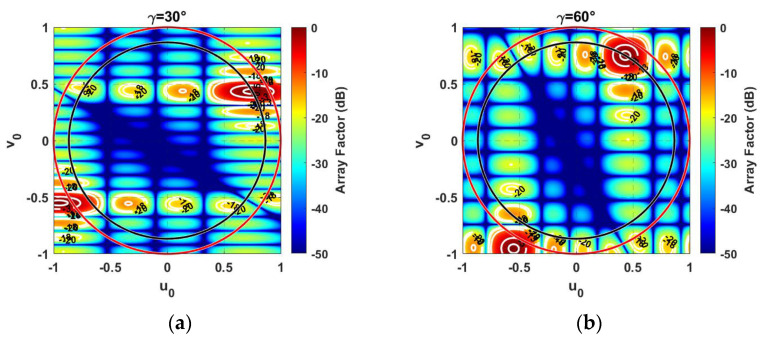
RP in the case of an antenna array with 8 × 8 isotropic elements arranged in a triangular lattice in the event of (**a**) γ = 30°, and (**b**) γ = 60°. The red circle represents the visible region, whereas the black circle highlights the circular sector cell.

It is apparent that the γ value has a considerable effect on *d*_0_. Specifically, γ = 30° and γ = 60° provide the largest minimum distance between elements, which is equal to 5.97 mm (0.587 λ_HF_) in the addressed scenario. Moreover, Figure 4 emphasizes how the triangular lattice provides the designer more degrees of freedom due to different possible combinations of element spacing (*d_x_*, *d_y_*). For example, let us consider the *RP* of an 8 × 8 array of isotropic elements (i.e., *E_nm_*(*θ,ϕ)* = 1) when the main beam is steered along one of the *ϕ* directions where there are grating lobes and when *θ* = 60°. Figure 5 illustrates the outcomes for two values of γ, namely, 30° and 60°. It is apparent that the different antenna element spacing (Figure 4) determines a different pattern shape, although the minimum distance remains the same. In particular, in the event of γ = 30° (Figure 5a), the antenna array presents a superior spatial resolution along the *v* plane, whereas the equilateral triangular lattice (γ = 60°) provides an almost circular footprint in the *u–v* plane. Moreover, in both cases the grating lobe is evidently outside the visible region (red circle), located at a distance of *R* = 1.1 from the center (*u* = 0, *v* = 0), as expected. Therefore, according to the desired spatial resolution in the *u–v* plane, it is possible to select the proper γ value.

In order to find the most suitable triangular lattice grid—and hence, the γ value—the angular average gain (*η_Gain_*), namely, the mean value of the gain attained during the beam steering inside the all-circular sector, in the case of a planar array composed of 8 × 8 elements, was evaluated as a function of the γ value within the addressed bandwidth (24.25–29.5 GHz), and by considering the beam scan within the circular sector cell, as shown in Figure 2. An element pattern shape equal to *E*(*θ*,*ϕ*) = *cos(θ)*, with a half-power beamwidth (HPBW) of 90°, was assumed as the element factor during the average gain evaluation.

**Figure 6:**
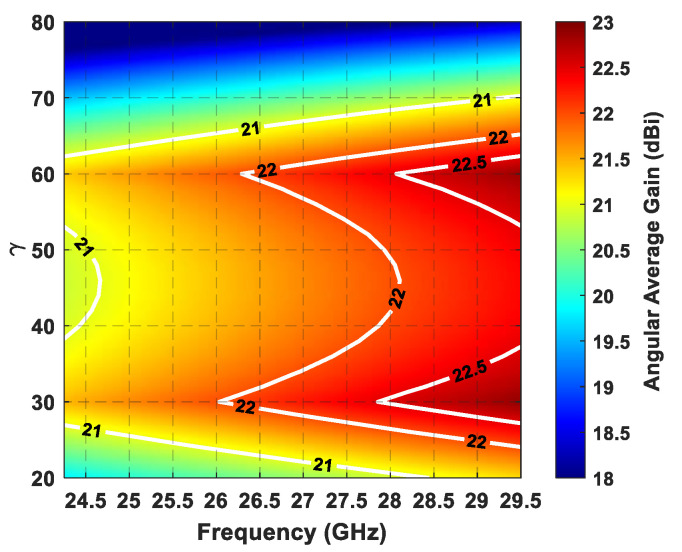
Angular average gain (*η_Gain_*) as a function of the γ value within the working frequencies (24.25 –29.5 GHz) in the case of a triangular lattice planar array of 8 × 8 elements.

From the color map of Figure 6 it can be concluded that *η_Gain_* always increases with frequency, although it exhibits higher value fluctuations with respect to the γ value, especially in the lower band. Furthermore, *η_Gain_* grows almost linearly up to γ = 30°, then gradually decreases with the increase of the γ value up to around γ = 45°, where a reversal of the trend occurs. Subsequently, the angular average gain reaches the second peak at γ = 60°, after which it quickly drops again. Therefore, by considering the minimum element spacing (Figure 4) and the angular average gain (Figure 6), it is possible to infer that with a circular sector cell there are two optimal γ values in cases of triangular grids, namely, γ = 30° and γ = 60°. In fact, these values ensure the largest minimum antenna element distance and, hence, the lowest performance degradation due to the elements’ mutual coupling [41,42], in addition to having the highest angular average gain as a function of the frequency. 

**Figure 7:**
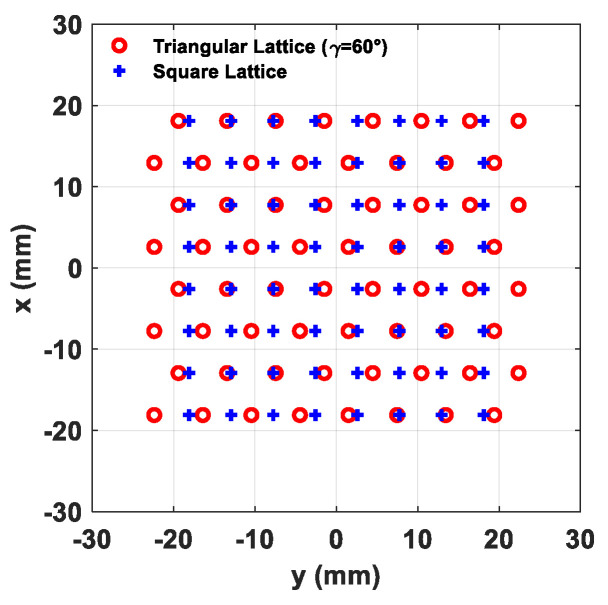
Array geometry of a planar array of 64 elements (8 × 8) in a triangular lattice (γ = 60°) and a square lattice.

In view of highlighting the impact of the antenna array lattice, two 8 × 8 planar arrays were considered—the former with elements arranged on a square lattice, and the latter on an equilateral triangular (γ = 60°) grid. It is worth noting that, as stated before, in a triangular lattice placement of the elements there are two most suitable values, namely, γ = 30° and γ = 60°. However, in the following performance comparison, it was decided to use an equilateral triangular lattice due to its more uniform footprint in the *u–v* plane, as shown in Figure 5b. By considering the Equations (3)–(5), with *R* = 1.1, for a square lattice *d*_0_ turns out to be 5.17 mm (0.508 λ_HF_), whereas the equilateral triangular grid exhibits a minimum element distance of 5.97 mm (0.587 λ_HF_), hence offering a 13.5% improvement. It is worth observing that values of *R* greater than 1 assure the absence of grating lobes during the beam steering inside the circular sector [43]. The same *R* value for both lattices provides the same distance in the *u–v* plane between the closest grating lobes and the center of the visible region (*u* = 0, *v* = 0), and hence, the same PSLL value as a function of the beam steering inside the circular sector cell. Furthermore, PSLL is around –13.3 dB at the broadside, whereas with the increase in the *θ* steering angle, PSLL degrades up to around –9 dB for *θ =* 60° due to the cosine-shaped elements’ radiation pattern. The larger minimum distance obtained via a triangular lattice arrangement allows us to achieve a lower level of mutual coupling among antenna elements by ensuring greater robustness of the active impedance of the array elements along the beam steering, a superior linearity of the employed power amplifiers (PAs), and a general improvement of the massive MIMO performance [41]. The wider element distance offered by the triangular lattice can be also considered advantageous for the thermal aspect, by helping the cooling system of the transceiver, which is critical in large mm-wave phased arrays [31]. The overlapped array geometry of triangular and square lattices is illustrated in Figure 7.

The larger array area of the triangular element arrangement also guarantees a better angular resolution and, in a massive MIMO scenario, a reduced angular interval within which users cannot be spatially resolved [2].

**Figure 8:**
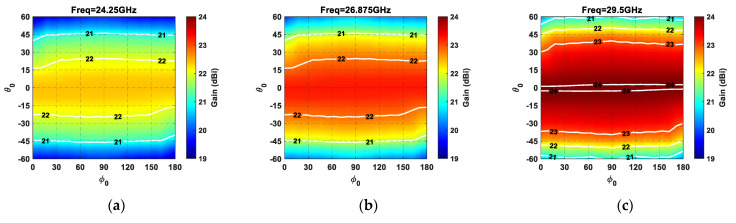

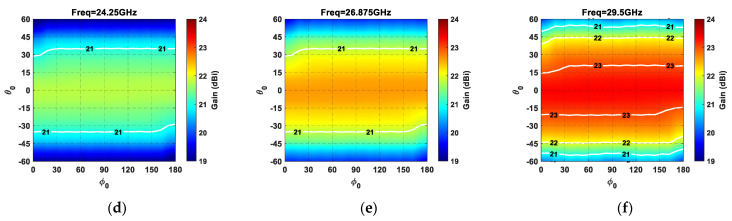
Gain in dBi of a planar array composed of 64 elements (8 × 8 array) as a function of the main beam steering inside the circular angular sector (−60° ≤ *θ_0_* ≤  60°*,* 0° ≤  *ϕ_0_* ≤  180°). Equilateral triangular lattice at (**a**) 24.25 GHz, (**b**) 26.875 GHz, and (**c**) 29.5 GHz; square lattice at (**d**) 24.25 GHz, (**e**) 26.875 GHz, and (**f**) 29.5 GHz.

The array gain as a function of the main beam direction (*θ_0_*, *ϕ_0_*) for both arrays of Figure 7 is illustrated in Figure 8 by considering an element pattern equal to *E*(*θ*,*ϕ*) = *cos(θ)*. It can be noted that, although the employed array lattices present similar trends during beam steering, the triangular lattice outperforms the square one due to a higher array gain. Furthermore, the gain value presents the highest value along the broadside (*θ_0_* = 0°), then decreases during the main beam steering due to beam widening and the approaching of the grating lobes inside the visible region [39]. For a more comprehensive analysis, the impact of the employed lattice on the array gain was also examined from a statistical point of view, by calculating the mean value (*η_Gain_*), the variance (*σ*^2^*_Gain_*), the minimum value (*min_Gain_*), and the maximum value (*max_Gain_*). The results reported in Table 1 emphasize that the triangular lattice enables the attainment of an average linear array gain improvement of around 13 %, when compared to a square lattice. Moreover, as visible from the color maps of Figure 8, the use of a triangular lattice provides both a superior minimum value (*min_Gain_*) and maximum value (*max_Gain_*) to those of a square grid.

**Figure 9:**
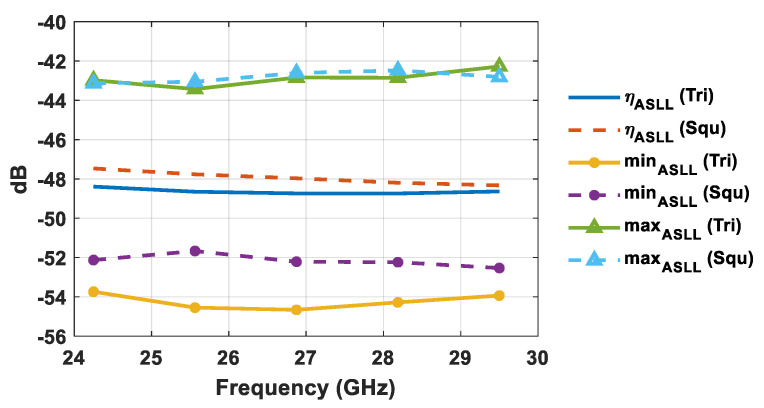
ASLL statistical comparison of square and equilateral triangular lattices by considering the whole visible region.

**Figure 10:**
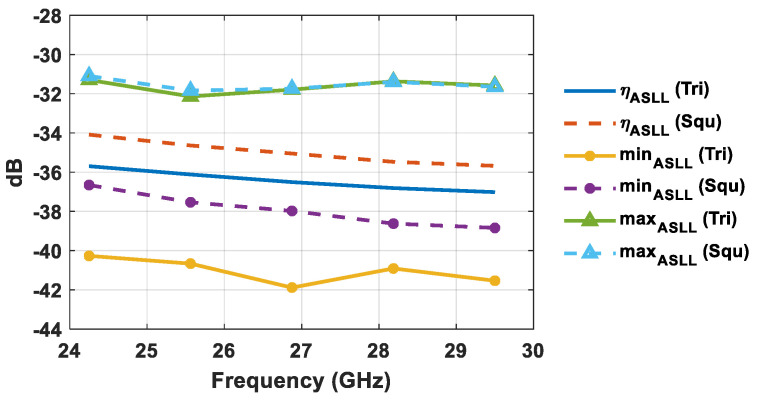
ASLL statistical comparison of square and equilateral triangular lattices by considering the circular sector cell.

Since the user’s interference plays a key role in massive MIMO systems, the impact of the employed array lattice on the average sidelobe level (ASLL) was evaluated from a statistical point of view. For the ASLL evaluation, the addressed region was selected for each main beam pointing at a user by the following ellipse equation:

(6) $$\begin{array}{l} \frac{\left(u-u_{0}\right)}{r_{u}^{2}}+\frac{\left(v-v_{0}\right)}{r_{v}^{2}}>1\\ r_{u}=1.2\frac{\lambda }{L_{x}}\;,\;r_{v}=1.2\frac{\lambda }{L_{y}} \end{array}$$

where *L_x_* and *L_y_* represent the array side length along the *x* and *y* directions; λ is the wavelength; (*u_0_,v_0_*) the desired direction in the *u–v* plane in which to steer the main beam; and *r_u_* and *r_v_* identify the main beam in the *u* and *v* planes, respectively. Once the sidelobe region has been selected for a desired (*u_0_,v_0_*) direction, the ASLL is calculated by averaging all of the array’s radiation pattern values inside the sidelobe region. Moreover, since the array can steer the main beam inside a circular area (Figure 2), we decided to evaluate the ASLL mean (*η_ASLL_*), minimum (*min_ASLL_*), and maximum (*max_ASLL_*) values by considering different scan angles (*N_θ_* = 15, *N_ϕ_* = 91) uniformly distributed inside the circular sector through the following equations:

(7) $$\begin{array}{l} \eta_{ASLL}=\frac{1}{N_{\theta }N_{\phi }}\sum_{i=1}^{N_{\theta }}\sum_{j=1}^{N_{\phi }}ASLL\left(i,j\right)\\ \min_{ASLL}=\min\left\{ASLL\left(i,j\right)\right\}\;,\;i=1,2,3,\ldots ,N_{\theta },j=1,2,3,\ldots ,N_{\phi }\\ \max_{ASLL}=\max\left\{ASLL\left(i,j\right)\right\}\;,\;i=1,2,3,\ldots ,N_{\theta },j=1,2,3,\ldots ,N_{\phi } \end{array}$$

where ASLL(*i*,*j*) represents the ASLL when the array main beam direction is toward the direction identified by (*i*,*j*). The calculated ASLL mean (*η_ASLL_*), minimum (*min_ASLL_*), and maximum (*max_ASLL_*) values are illustrated in Figure 9 and Figure 10. Specifically, Figure 9 highlights the ASLL statistical comparison in the event that the sidelobe region is represented by the intersection between Equation (6) and the *u–v* points inside the unit radio’s circle, whereas the ASLL assessment of Figure 10 takes into account a sidelobe region represented by the intersection between Equation (6) and the *u–v* points inside the circular sector cell shown in Figure 2. 

By looking at Figure 9 and Figure 10 it is evident that the use of triangular lattices in planar antenna arrays outperforms that of square ones from a statistical point of view, in terms of ASLL. The better performance of triangular lattices is highlighted by a lower ASLL mean value, a reduced minimum value, and a similar maximum value. Specifically, triangular lattices allow for a percentage reduction of the ASLL mean value (*η_ASLL_*) of 1–2% within the desired bandwidth, compared to square lattices, when considering the whole visible region. The ASLL superiority of triangular lattices is further confirmed by taking into account only the investigated circular sector cell (Figure 10). Indeed, the ASLL mean value percentage reduction in triangular lattices compared to square lattices turns out to be between 3.5% and 4.5%. This feature is attractive for 5G massive MIMO systems, since it allows for a reduction in both the intra-cell and inter-cell interference. Since lateral lobes related to the main beam pointing at one user generate interference for all other users served within the same time–frequency resource, the lower ASLL guaranteed by a triangular lattice is appealing for use in a HAPS 5G massive MIMO system.

## 3. Phased Array Comparison of Triangular and Square Lattices

In this section, a planar array composed of 64 elements (8 × 8) is analyzed to better emphasize the advantages of using a triangular lattice. The full-wave electromagnetic simulations were carried out using Ansys HFSS [44]. Each single element consists of a square patch antenna printed on a 0.7 mm thick grounded dielectric layer (RO5880), fed through a coaxial cable. As mentioned in the previous section, the minimum antenna element spacing for a square lattice is 5.17 mm (0.508 λ_HF_), whereas the equilateral triangular grid provides a minimum element distance of 5.97 mm (0.587 λ_HF_).

**Figure 11:**
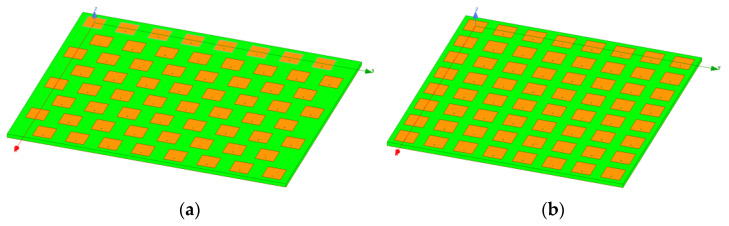
Planar array with 64 elements arranged with (**a**) an equilateral triangular lattice; and (**b**) a square lattice.

Since antenna array design is beyond the scope of this paper, the lattice comparison as a function of frequency was pursued by tuning the antenna array elements to be matched in one narrowband frequency band at time. The finite array with 64 elements, with the antenna elements tuned to 29.5 GHz, is illustrated in Figure 11. Specifically, the *x–z* plane represents the E-plane of the array, whereas *y–z* represents the H-plane. At the beginning, the mutual coupling (*S_ij_*) among antenna elements was addressed. The simulated mutual coupling average value (*η_Sij_*) among antenna elements, and the maximum mutual coupling as a function of the frequency, are illustrated in Table 2. It can be seen that a triangular lattice enables us to considerably reduce both the mutual coupling average value and the maximum mutual coupling. For instance, the peak mutual coupling achieved at the highest frequency (29.5 GHz) with a square lattice (−14.4 dB) turns out to be worse than the peak mutual coupling achieved at the lowest frequency (24.25 GHz) for the triangular lattice planar array (−14.91 dB). In general, the mutual coupling difference between triangular and square lattices is more pronounced at the lowest frequencies, and then the coupling difference tends to reduce with the increase in frequency. 

**Figure 12:**
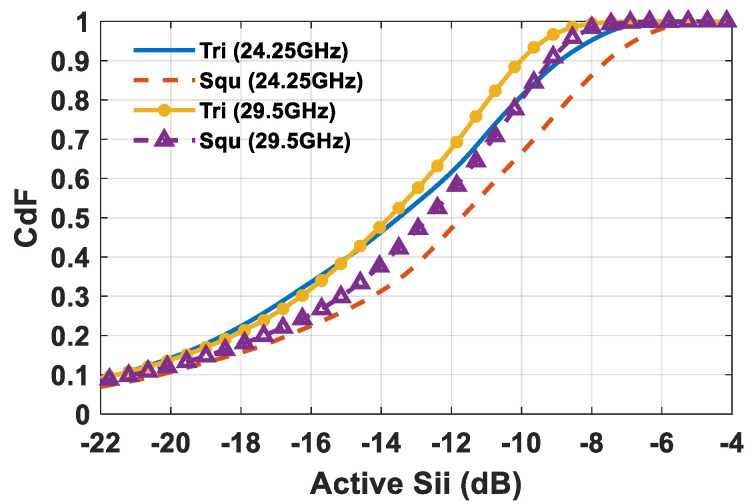
Active *S_ii_* CDF for square and equilateral triangular lattice planar arrays composed of 64 elements at 24.25 and 29.5 GHz.

One of the detrimental effects of mutual coupling is the variation of the antenna elements’ input impedance during beam steering. The impact of the employed array lattice on active *S_ii_* parameters (*i* = 1,…,64) was evaluated from a statistical point of view through the cumulative distribution function (CDF) of the active *S_ii_* of all of the antenna array elements during the coverage. From Figure 12, a better statistical behavior of active *S_ii_* parameters in triangular lattice than in square one is clearly recognizable. Indeed, the triangular lattice, thanks to a lower mutual coupling (Table 2), provides an average value of the active *S_ii_* of around 13.8 dB within the investigated frequencies, whereas the square one is characterized by a mean value between −11.8 dB and −12.5 dB. 

To further emphasize the superior robustness of the antenna elements’ impedance variation when using a triangular lattice, the simulated array matching efficiency (*η*_Γ_) was evaluated for all of the investigated steering angles by, using the following equation:

(8) $$\eta_{\Gamma }=\frac{\sum_{i=1}^{64}\left(1-{\left|S_{ii}\right|}^{2}\right)}{64}$$

**Figure 13:**
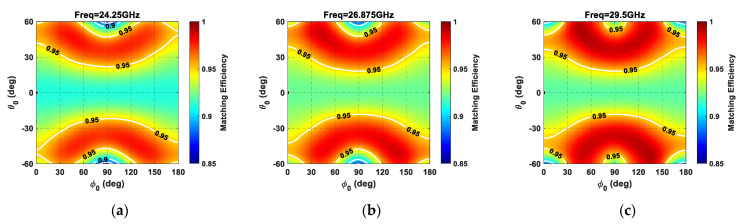

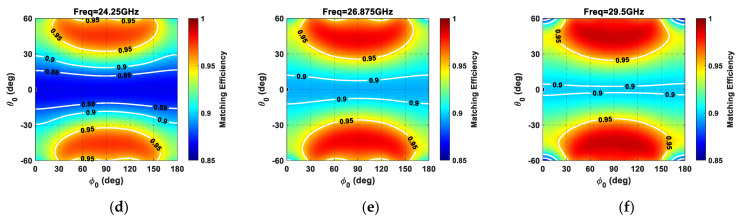
Matching efficiency (*η*_Γ_) of a planar array composed of 64 elements (8 × 8) as a function of the main beam steering inside the circular angular sector (−60° ≤ *θ_0_* ≤  60°*,* 0° ≤  *ϕ_0_* ≤  180°). Equilateral triangular lattice at (**a**) 24.25 GHz, (**b**) 26.875 GHz, and (**c**) 29.5 GHz; square lattice at (**d**) 24.25 GHz, (**e**) 26.875 GHz, and (**f**) 29.5 GHz.

The color maps of Figure 13 confirm the advantages of adopting triangular lattices in planar arrays, rather than square ones, in that they provide higher efficiency values for all investigated frequencies (24.25–29.5 GHz). Specifically, the triangular lattice provides a higher matching efficiency value than the square lattice for all of the investigated steering angles, except around *ϕ* = 90° plane (H-plane of the array) for *θ* angles greater than 40°. In particular, the triangular lattice shows a better matching efficiency in approximately 90% of the covered area within the addressed frequency band.

**Figure 14:**
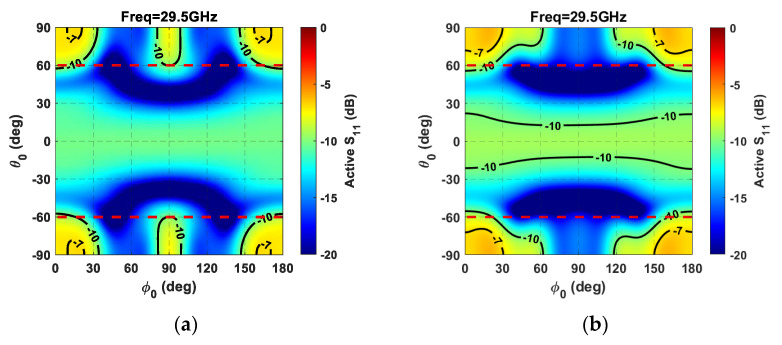
Active *S_11_* parameter related to the central array element as a function of beam steering for a planar array composed of 64 elements with (**a**) a triangular lattice; and (**b**) a square lattice.

The main reason the triangular lattice’s matching efficiency undergoes a value reduction in the principal planes for *θ* angles close to 60° at the highest frequency is due to scan blindness onset. This phenomenon can be observed through the active *S_11_* parameter related to the array’s central element at the highest frequency (29.5 GHz), as a function of the beam steering shown in Figure 14. To emphasize the scan blindness onset, the active *S_11_* parameter was considered during beam steering within the whole visible region. More in detail, the color maps highlight that the triangular lattice does not avoid the scan blindness onset—characterized by a strong antenna element mismatch—but only reduces it by pushing the involved angular sector a bit further [43]. In fact, by considering the simulated phased array in the event of beam steering, the square lattice provides an active *S_11_* higher than –10dB within the angular ranges |*θ_0_*| > 60°, *ϕ_0_* < 60°, and *ϕ_0_* > 120° (Figure 14b). On the other hand, the triangular lattice presents a milder mismatch of the central element for |*θ_0_*| > 60°, *ϕ_0_* < 30°, *ϕ_0_* >150°, and 80°< *ϕ_0_* < 100° (Figure 14a). Moreover, by considering the angular sector with |*θ_0_*| > 60°—hence, outside the desired scan angle area (highlighted by dashed red lines)—the triangular lattice presents an *S_11_* central array element higher than −10dB in 40 % of cases, whereas the square lattice does so in 50 % of cases.

**Figure 15:**
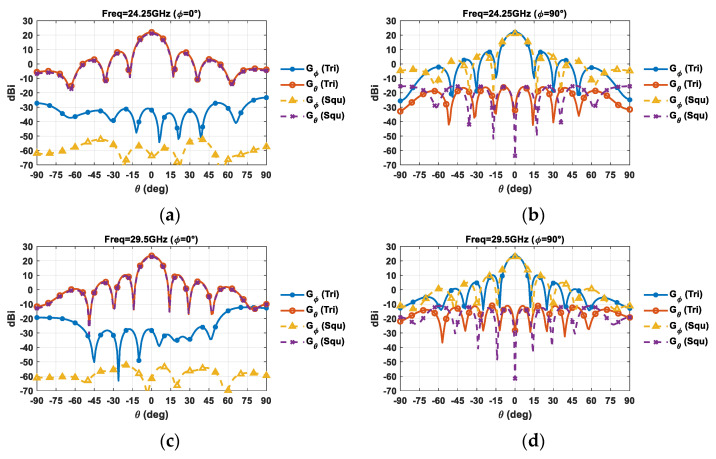
Simulated realized gain for a planar array composed of 64 elements at a broadside direction: (**a**) 24.25 GHz *ϕ* = 0°; (**b**) 24.25 GHz *ϕ* = 90°; (**c**) 29.5 GHz *ϕ* = 0°; and (**d**) 29.5 GHz *ϕ* = 90°.

With the aim of evaluating the impact of the antenna array lattice on the radiation pattern shape, the realized gain as a function of the *θ* angle at a broadside direction is reported in Figure 15.

Figure 15 confirms that a triangular lattice provides a narrower main beam in the *ϕ* = 90° plane, thus providing a higher maximum gain and better angular resolution. Moreover, a triangular lattice leads to comparable cross-polar levels to square one in the *ϕ* = 90° plane, whereas it introduces a slight degradation in the E-plane of the array (Figure 15a,c)—although a cross-polar level of about 50 dB around the mean beam is still guaranteed.

## 4. Massive MIMO Performance Evaluation

The comparison of triangular and square lattices was carried out in terms of array gain, ASLL, active *S_ii_* parameters, and matching efficiency, by considering a circular sector in which to serve users. However, one of the key factors that will allow us to drastically improve the data throughput of the upcoming 5G wireless technology will be the use of massive MIMO technology capable of serving different users within the same time–frequency resource through multibeam radiation patterns. In general, various beamforming methods are available for the achievement of a multibeam system [10,45,46].

Let us consider a HAPS equipped with an array of *M* = 64 (8 × 8) antenna elements that serves *K* concurrent ground users, equipped with a single isotropic antenna, located inside the above-mentioned circular angular sector (Figure 2), through a multibeam radiation pattern in a line-of-sight (LOS) scenario. The assumption of a LOS channel is consistent since, at mm-wave, the LOS ray represents the predominant mode of propagation between the base station (BS) and the users, due to large path loss as well as the use of high-gain antennas [47,48]. Furthermore, the LOS channel condition turns out to be further emphasized in the case of HAPS wireless communication [49]. 

The received signal-to-interference-plus-noise ratio (SINR) for the *n^th^* user in case of a multi-user scenario can be written as [40,50,51]:

(9) $$SINR_{n}=\frac{\delta \eta_{\Gamma }\left(\theta_{n},\phi_{n}\right)G\left(\theta_{n},\phi_{n}\right)}{\delta \sum_{i=1}^{K}G\left(\theta_{i},\phi_{i}\right){\left|h_{i}*W_{n}\right|}^{2}+1}$$

where *G*(*θ_n_*,*ϕ_n_*) represents the HAPS array’s gain toward the *n^th^* user located at (*θ_n_*,*ϕ_n_*); *δ* represents the ratio between the transmitted power (*P_t_*) and the noise power (*σ_0_*); *h_n_* ϵ 1×*M* (*n* = 1,2,..,*K*) corresponds to the channel propagation between the *n^th^* user and the HAPS antenna array; and *w_n_* ϵ *M*×1 consists of the precoding vector that, in general, depends on the selected beamforming method. It is worth noting that, differently from Reference [37], the simulated matching efficiency (*η*_Γ_) was included in (9) in order to achieve a more accurate SINR estimate. To evaluate the system quality of service, a maximum ratio (MR) precoding technique [2] with a perfect channel state information (CSI) was assumed in the following analysis. However, it is worth noting that more efficient precoding and combining algorithms able to reduce interference could be exploited [52] but, in general, they require more complexity and turn out to be more sensitive to channel estimation error. Additionally, since array elements’ mutual coupling (MC) undermines the MIMO performance [53] due to the unwanted correlation among elements, the channel matrix *H* is modelled as [54]:

(10) $$H=H_{0}\left(I_{mxm}-SS^{H}\right)$$

where *H_0_* ϵ *K* × *M* denotes the complex channel gain among the users and the HAPS antenna elements in the absence of antenna mutual coupling; *I_m×m_* is an identity matrix; and *S* ϵ *M*×*M* corresponds to the scattering parameters matrix of the HAPS planar array. Unlike [37], the channel matrix was evaluated by taking into account in (10) the full-wave simulation of the planar array, and not just the antenna element distance. Once the SINR is known, the maximum achievable bitrate over 1 Hz of bandwidth for the *n^th^* user, which is the spectral efficiency (SE) per user, is:

(11) $$SE_{n}=\log_{2}\left(1+SINR_{n}\right)$$

**Figure 16:**
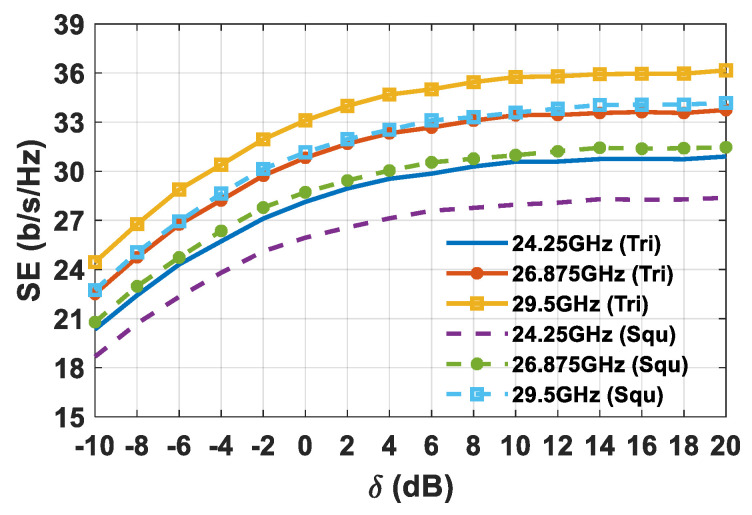
SE comparison for a planar array composed of 8 × 8 elements, in cases of square and triangular lattices, as a function of the *δ* ratio in dB in the event of 8 concurrent users.

For the SE assessment both the simulated array gain and the *S*-matrix of the planar arrays shown in Figure 11 were used. Furthermore, 10,000 sets of *K*-concurrent users randomly distributed inside the circular sector were adopted. It is worth observing that a smart user’s selection inside the sector can improve the massive MIMO performance and the energy efficiency of the wireless communication [55]. However, this aspect was neglected, since the main goal of the paper is to highlight the performance differences between square and triangular lattices. The achievable SE as a function of the *δ* value, which is the ratio between the transmitted power (*P_t_*) and the noise power (*σ_0_*), is reported in Figure 16 for both square and triangular lattices in the event of eight simultaneous users inside the investigated circular sector.

**Figure 17:**
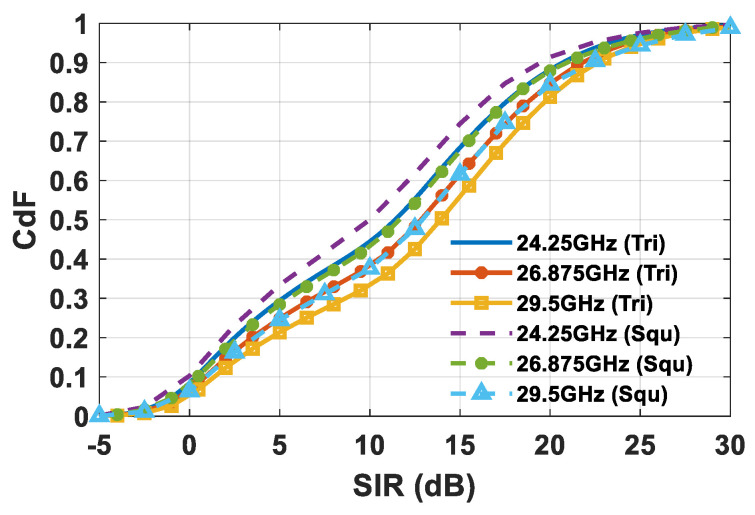
SIR CDF comparison for a planar array composed of 8x8 elements, in cases of square and triangular lattices, in the event of 8 concurrent users.

It can be seen that the triangular lattice deployment of the array antenna elements allows for the achievement of a considerable improvement in the SE. The SE starts to grow almost linearly with the increase in the *δ* value, after which it reaches a superior limit due to the SIR, and the system becomes interference limited. Therefore, once the upper SE boundary is reached, further increasing the transmitted power does not represent an efficient way of improving the SE. The improved SE in the triangular lattice array is apparent from looking at the statistical behavior of the CDF of the SIR (Figure 17), and both the average SIR (*η_SIR_*) and 90% of the SIR occurrence (*SIR_90%_*), as highlighted in Table 3.

**Figure 18:**
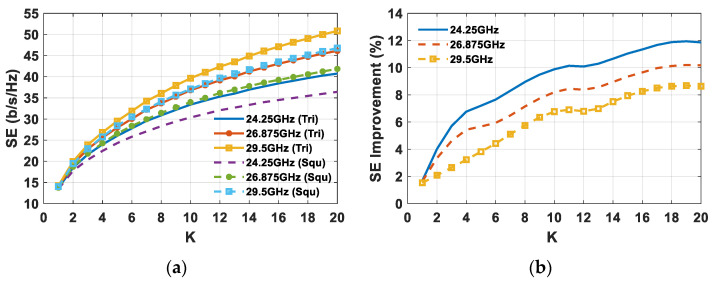
(**a**) SE comparison as a function of the number of users (*K*) between square and triangular lattice planar arrays composed of 64 elements; and (**b**) percentage SE improvement in the event of *δ* = 20 dB.

Since to improve the SE it is better to serve more users inside the predefined sector cell rather than increasing the transmitted power, the SE is plotted as a function of the number of users (*K*) for both triangular and square lattices in the event of a *δ* = 20 dB (Figure 18).

The remarkable advantage of using a triangular lattice arrangement of the array elements instead of a square one is confirmed by observing the SE in Figure 18a. To emphasize the SE comparison, the percentage SE improvement is calculated and reported in Figure 18b. As can be seen, the achievable SE between the two lattices turns out to be comparable for few users, even if the triangular lattice’s SE is somewhat higher due to a superior array gain value (Figure 8). Conversely, with the increase in the number of concurrent users, the SE improvement guaranteed by the triangular lattice array grows continuously, and reaches a value between 8.5% and 12 % in the event of 20 simultaneous users inside the sector.

In view of highlighting the advantageous impact of the triangular lattice on planar arrays in massive MIMO systems, the percentage SE improvement of the most suitable triangular lattice, compared to rectangular or square lattices, is reported in Table 4 for a case of 20 concurrent users deployed inside the different sector cells. Table 4 confirms that, although with some performance differences, the triangular lattice turns out to be the most efficient array grid regardless of the sector cell or the adopted frequency within the considered range. It is worth observing that, in practice, a minimum user distance within the same time–frequency resource is required in order to avoid high SIR and, hence, increase the SE. However, SE improvement between triangular and square lattices remains substantially unchanged. A further analysis aimed at understanding the role of array gain and the elements’ mutual coupling (MC) was performed. The SE improvement between triangular and square lattices, as shown in Figure 18b, was recalculated under the hypothesis of having the same array gain (or the same received signal) for both of the employed lattices (20 dBi for all the frequencies), and in the case of an ideal array without MC (w/o MC).

**Figure 19:**
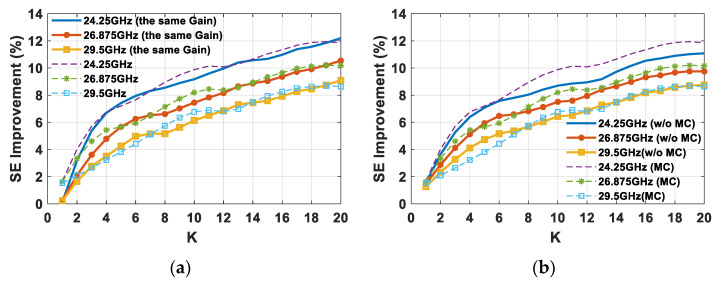
Percentage SE improvement as a function of the number of users (*K*) between square and triangular lattice planar arrays composed of 64 elements in the event of (**a**) the same HAPS array gain for all of the frequencies for both lattices; and (**b**) an ideal planar array equipped with 64 elements without MC (w/o MC).

Figure 19a highlights how the higher array gain values, as a function of the steering angles obtained in the case of the triangular lattice planar array (Figure 8), allow for an increase in the SE for just a few users (up to three). In fact, the SE improvement with the same array gain in the case of just a single user is equal to zero at all frequencies, whereas by increasing the number of concurrent users the curves are overlapped to the case of different gain values. Therefore, the higher SE provided by the triangular lattice in the event of many users is due to the better interference robustness guaranteed by the superior angular resolution. The SE improvement comparison of Figure 19b emphasizes how the MC effects contribute to a further increase in the percentage SE improvement of the triangular lattice compared to the square lattice, mainly at the lowest frequency (24.25 GHz). Conversely, the MC effect on percentage SE improvement gradually vanishes as the frequency increases, and becomes irrelevant at the highest frequency (29.5 GHz).

## 5. Conclusions

An extensive analysis of HAPS massive MIMO systems employing triangular lattices has been presented within the 5G NR n257 and n258 frequency bands (24.25–29.5 GHz). The noteworthy massive MIMO performance improvement when adopting a triangular lattice arrangement of the array elements instead of a square one in a planar array with a regular lattice has been highlighted. Specifically, a triangular lattice allows for the achievement of a superior array gain and ASLL reduction, as well as greater robustness of the antenna elements’ impedance variation during beam steering by exploiting lower MC levels. Moreover, the larger minimum distance between elements in the case of a triangular grid guarantees a better angular resolution that, in a massive MIMO scenario, provides superior interference robustness and, hence, higher SE. The advantages offered by the regular and periodic triangular lattice arrangement of antenna elements make it appealing for 5G massive MIMO applications.

## Figures and Tables

**Table 1:** Statistical comparison between the gain (dBi) of a square lattice and an equilateral triangular lattice for an 8 × 8 array evaluated within the circular scan sector.

	24.25 GHz		26.875 GHz		29.5 GHz	
	Tri	Squ	Tri	Squ	Tri	Squ
***η_Gain_***	21.38	20.77	22.18	21.57	22.87	22.28
***σ^2^_Gain_***	0.89	0.83	0.97	0.9	1.12	0.99
***min_Gain_***	19.17	18.73	19.81	19.38	20.24	19.83
***max_Gain_***	22.42	21.78	23.26	22.61	24.03	23.37

**Table 2:** Simulated mutual coupling average values (*η_Sij_*) among antenna elements and the maximum value comparison in dB between triangular and square lattice planar arrays composed of 64 elements (8 × 8).

	24.25 GHz		26.875 GHz		29.5 GHz	
	Tri	Squ	Tri	Squ	Tri	Squ
***η*** *_Sij_*	−48.6	−42.52	−49.35	−44.8	−50.74	−46.2
**Max(*S_ij_*)**	−14.91	−11.84	−15.62	−12.55	−16.9	−14.4

**Table 3:** Simulated average SIR (*η_SIR_*) in dB and *SIR_90%_* across triangular and square lattice planar arrays composed of 64 elements (8 × 8), in the event of 8 served users inside the circular sector.

	24.25 GHz		26.875 GHz		29.5 GHz	
	Tri	Squ	Tri	Squ	Tri	Squ
***η*** ***_SIR_***	11.1	10	12	11.25	13.3	12.2
***SIR_90%_***	0.4	0	0.9	0.5	1.4	1

**Table 4:** Percentage SE improvement offered by the most suitable triangular lattice, compared to rectangular/square lattices, in the case of planar arrays composed of 64 elements (8 × 8) for 20 served users inside the different sector cells.

	Sector	Lattice	SE Improvement	
			24.25 GHz	29.5 GHz
[37]	Rectangular	γ = 36.6° vs Rect	14 %	10 %
This Paper	Circular	γ = 60° vs Squ	12 %	8.5 %

## Data Availability

Not applicable.
